# The Role of Acceptance and Commitment Therapy in Improving Social Functioning Among Psychiatric Patients: A Systematic Review

**DOI:** 10.3390/healthcare13131587

**Published:** 2025-07-02

**Authors:** Desirèe Latella, Giulia Marafioti, Caterina Formica, Andrea Calderone, Elvira La Fauci, Angela Foti, Rocco Salvatore Calabrò, Giuseppa Filippello

**Affiliations:** 1 Giulia Marafioti, Psy, IRCCS Centro Neurolesi Bonino-Pulejo, S.S. 113 Via Palermo, C.da Casazza, 98124 Messina, Italy; desiree.latella@irccsme.it (D.L.); katia.formica@irccsme.it (C.F.); andrea.calderone@irccsme.it (A.C.); roccos.calabro@irccsme.it (R.S.C.); 2 Department of Clinical and Experimental Medicine, University of Messina, Piazza Pugliatti, 1, 98122 Messina, Italy; elvira.lafauci@gmail.com (E.L.F.); angela-foti@alice.it (A.F.); giuseppa.filippello@unime.it (G.F.)

**Keywords:** acceptance and commitment therapy, psychiatric disorders, social functioning

## Abstract

Background and Objectives: Acceptance and commitment therapy (ACT) enhances psychological flexibility by fostering acceptance of thoughts and emotions, promoting mindfulness practices, and encouraging engagement in value-based actions. These processes have been associated with improvements in mental health and social functioning, with accumulating evidence supporting ACT’s efficacy across various psychiatric disorders. This systematic review aimed to evaluate current evidence on ACT interventions for reducing psychiatric symptoms and enhancing social functioning and interpersonal relationships in adults with psychiatric conditions. Materials and Methods: A comprehensive search was conducted across PubMed, Web of Science, the Cochrane Library, and Embase for studies published between 2014 and 2024. The review protocol was registered on the Open Science Framework (OSF; registration ID: 2ZAGT). Results: Seventeen studies met the inclusion criteria; however, the evidence base remained fragmented, with most psychiatric diagnoses represented by only one or two studies. The reviewed studies exhibited several methodological limitations, including small sample sizes, lack of randomization and blinding, high risk of bias, reliance on subjective outcome measures, and inadequately designed or absent control groups. Despite these limitations, ACT was associated with beneficial effects in conditions such as post-traumatic stress disorder (PTSD), insomnia, psychosis, and autism spectrum disorder, particularly in reducing experiential avoidance, enhancing mindfulness, and promoting long-term improvements in emotional regulation and life satisfaction. Conclusions: Due to the limited number of studies per diagnosis, significant methodological weaknesses, and the lack of high-quality controlled trials, this review cannot provide strong evidence for the efficacy of ACT in improving social functioning among adults with psychiatric disorders. The heterogeneity and overall low quality of the available literature highlight the urgent need for further large-scale well-controlled studies.

## 1. Introduction

### Social Impairment in Psychiatric Disorders

According to DSM-5, mental disorders significantly affect individuals’ quality of life, as well as their cognitive, emotional, and behavioral functioning [1]. Although genetic and biological factors contribute to their development, environmental factors, such as stress and interpersonal relationships, play a crucial role in determining their course as risk factors or protective factors [2,3]. A common consequence of many psychiatric disorders is social impairment, characterized by difficulties in building and maintaining interpersonal connections, which often exacerbates the underlying condition [4,5]. Social functioning is the ability to effectively engage with and adapt to social and environmental demands [6]. This complex integration of cognitive, emotional, and behavioral processes is often impaired in conditions such as anxiety, depression, schizophrenia, and chronic pain [7,8,9,10]. These impairments are frequently overlooked until the psychiatric condition has fully developed, compounding the challenges faced by affected individuals. Addressing social reintegration is, therefore, a critical component of effective therapeutic approaches, as social relationships play a fundamental role in overall well-being [11,12]. Traditional treatments for psychiatric disorders, such as cognitive-behavioral therapy (CBT) and social skills training, have focused on improving social functioning by restructuring maladaptive thought patterns and enhancing social interaction skills [13,14,15,16,17]. However, there is a growing interest in innovative approaches that address the psychological flexibility underlying social impairments. Acceptance and commitment therapy (ACT), developed by Hayes, Wilson, and Strosahl, is a behavioral therapy based on psychological flexibility. ACT emphasizes accepting rather than avoiding difficult emotions and thoughts, mindfulness, and engaging in behaviors aligned with personal values [18,19]. This approach encourages individuals to engage more fully with the present moment, enabling them to navigate life’s challenges with greater adaptability while fostering long-term well-being [20]. A core element of ACT is acceptance, which involves developing an open stance toward distressing emotions and thoughts. Rather than suppressing or avoiding these experiences, individuals are encouraged to acknowledge them without judgment [21]. This approach reduces the energy in fighting emotions, allowing individuals to focus on meaningful pursuits [22]. Acceptance promotes resilience by helping individuals view discomfort as a natural part of life rather than a barrier to progress. To complement acceptance, ACT introduces cognitive defusion, a strategy aimed at changing the individual’s relationship with their thoughts [23,24,25]. In ACT, thoughts are not seen as commands or truths but as fleeting mental events. Techniques such as visualizing a troubling thought as a leaf floating down a stream or repeating it aloud until it loses its emotional weight help individuals disentangle themselves from unhelpful cognitive patterns [26,27]. This process empowers individuals to respond to their thoughts with flexibility and creativity rather than reacting out of fear or habit. Mindfulness further strengthens psychological flexibility by promoting present and focused non-judgmental awareness [28,29,30]. By encouraging individuals to fully experience the present moment (whether through observing their breath, noting bodily sensations, or attentively engaging with their environment), ACT helps cultivate clarity and calmness. Mindfulness fosters an ability to pause and reflect, enabling individuals to respond thoughtfully rather than impulsively to challenging situations [31,32]. Central to ACT’s efficacy is its focus on value clarification, which involves helping individuals identify the principles and aspirations that matter most to them. This process guides individuals in uncovering what gives their life meaning, whether it is fostering relationships, pursuing professional goals, or contributing to a cause [33,34]. Once these values are identified, ACT supports individuals in taking committed action, encouraging them to persist in value-driven behaviors despite obstacles or discomfort. This alignment of actions with deeply held values often leads to a renewed sense of purpose and fulfillment [35,36]. ACT offers a holistic approach that enhances social connections and personal growth. It helps individuals shift from a cycle of avoidance and fear to one of acceptance and meaningful engagement, promoting resilience and adaptability in the face of life’s challenges [37]. Studies also highlight its impact on altering behaviors and enhancing well-being in individuals recovering from conditions like colorectal cancer [38,39,40]. By addressing psychological and behavioral patterns underlying social impairments, ACT promotes interpersonal growth and fosters stronger connections within diverse populations [41,42]. However, despite growing evidence for traditional treatments such as CBT and social skills training, these approaches often fall short in addressing the experiential avoidance and psychological inflexibility that contribute to long-term social dysfunction. Social impairments are often multifaceted and resistant to symptom-focused interventions, highlighting the need for therapies that integrate cognitive, emotional, and behavioral change mechanisms. The primary objective of this systematic review is to evaluate the effectiveness of acceptance and commitment therapy (ACT) in improving social functioning among adults with psychiatric disorders. Secondary objectives include assessing the impact of ACT on psychological flexibility, emotional regulation, and the quality of interpersonal relationships, and evaluating whether ACT demonstrates differential efficacy across various psychiatric diagnoses. This systematic review examines the evidence supporting ACT’s effectiveness in improving social functioning among adults with psychiatric disorders. By focusing on ACT’s unique processes, this review aims to highlight its potential as a therapeutic tool for addressing social impairments and guiding clinical practice and future research.

## 2. Materials and Methods

This review focused on evaluating the efficacy of acceptance and commitment therapy (ACT), rather than its effectiveness, to provide a clearer understanding of the intervention’s potential under controlled experimental conditions. Given the primary aim of this work—to assess the core psychological processes targeted by ACT and their association with specific psychiatric outcomes—we prioritized studies designed to test causal mechanisms, typically conducted in structured research settings. While some included studies that reflect real-world implementations, the emphasis on efficacy was necessary to reduce variability introduced by contextual and delivery factors and to better isolate the theoretical components of ACT responsible for change. This approach allows for a more rigorous assessment of ACT’s therapeutic impact across psychiatric diagnoses, laying the groundwork for future investigations into its effectiveness in routine clinical practice.

### 2.1. Inclusion Criteria

The inclusion criteria were as follows: (i) patients affected by psychiatric disorders (e.g., depression, psychosis, PTSD, anxiety); (ii) randomized clinical trials (RCT), pilot studies, and observational studies; (iii) studies published between 2014 and 2024; (iv) studies using ACT as the main therapeutic intervention; (v) studies assessing social functioning or related domains (e.g., social support, psychological flexibility, interpersonal functioning); (v) English language; and (vi) published in a peer-reviewed journal.

### 2.2. Exclusion Criteria

The exclusion criteria were as follows: (i) studies that combined ACT with other treatments without isolating ACT-specific effects; (ii) studies involving mixed or heterogeneous populations not limited to individuals with psychiatric diagnoses; (iii) studies with insufficient design to determine causal efficacy (e.g., retrospective, case reports, narrative reviews); (iv) studies focused on non-psychiatric populations (e.g., chronic medical illnesses without psychiatric diagnosis); (v) studies involving psychiatric disorders in children and adolescents (age < 18 years); (vi) studies that reported insufficient methodological detail to assess efficacy or outcome relevance.

### 2.3. PICO Evaluation

We utilized the PICO (population, intervention, comparison, outcome) model to establish our search terms. The population focused on adults diagnosed with psychiatric disorders, including anxiety, depression, PTSD, insomnia, psychosis (including early and complex presentations), autism spectrum disorders, and treatment-resistant or comorbid conditions. The intervention included all studies using the ACT approach, delivered in individual, group, or digital formats. The comparison involved analyzing psychotherapeutic interventions based on ACT and other conventional treatments, such as CBT, psychoeducation, communication training, and treatment-as-usual. The outcomes included any measurable improvements in psychological symptoms (e.g., depression, anxiety, hallucinations), as well as data on social functioning, quality of life, interpersonal relationships, and psychological flexibility. Table 1 provides a visually and organized summary of the PICO framework and study inclusion criteria.

### 2.4. Search Strategy

This systematic review employed a structured search strategy based on the PICO framework and was conducted between the 5 June 2024 and the 31 July 2024. The search was limited to studies published between 2014 and 2024 in order to capture the most relevant and recent evidence on the efficacy of acceptance and commitment therapy (ACT) in improving social functioning among adults with psychiatric disorders. The studies were identified by searching PubMed, Web of Science, Cochrane Library, and Embase databases. These platforms were selected for their complementary scope and methodological rigor: PubMed was added because it has the broadest and most comprehensive indexing of biomedical and psychological literature, which is particularly pertinent for identifying studies of mental health interventions and clinical outcomes. Web of Science was selected because of its multidisciplinary access and its strong citation indexing capacities, which enable research that is shaping and being shaped by both the psychological and psychiatric fields to be identified. The Cochrane Library was chosen because of its focus on high-quality systematic reviews and randomized controlled trials, which provide the best evidence on the efficacy of treatment interventions. Finally, we searched Embase (due to its depth in clinical, drug, and psychiatric research content) to ensure a complete search across peer-reviewed studies on ACT and psychosocial functioning. Although the search strategy did not undergo a formal PRESS review, it was internally peer-reviewed by two authors with experience in systematic reviews and database searching to ensure clarity, comprehensiveness, and methodological soundness. The full Boolean search string used in PubMed was as follows:

(“mental disorders”[MeSH Terms] OR (“mental”[All Fields] AND “disorders”[All Fields]) OR “mental disorders”[All Fields] OR (“psychiatric”[All Fields] AND “disorder”[All Fields]) OR “psychiatric disorder”[All Fields]) AND (“acceptance and commitment therapy”[MeSH Terms] OR (“acceptance”[All Fields] AND “commitment”[All Fields] AND “therapy”[All Fields]) OR “acceptance and commitment therapy”[All Fields]) AND (“social interaction”[MeSH Terms] OR (“social”[All Fields] AND “interaction”[All Fields]) OR “social interaction”[All Fields] OR (“social”[All Fields] AND “functioning”[All Fields]) OR “social functioning”[All Fields])

Equivalent search terms were adapted to the syntax requirements of each database. Core terms included variations of psychiatric disorders, ACT-based interventions, and outcomes related to social functioning and interpersonal relationships. A summary table of the core keywords used across databases is provided in the Supplementary Material. This multistep approach ensured a comprehensive, reproducible, and methodologically robust identification of relevant studies assessing the impact of ACT on psychological symptoms and social outcomes in psychiatric populations. Table 2 provides the PICO elements and the keywords/terms used for the search stategy.

#### 2.4.1. Data Extraction

**Figure 1:**
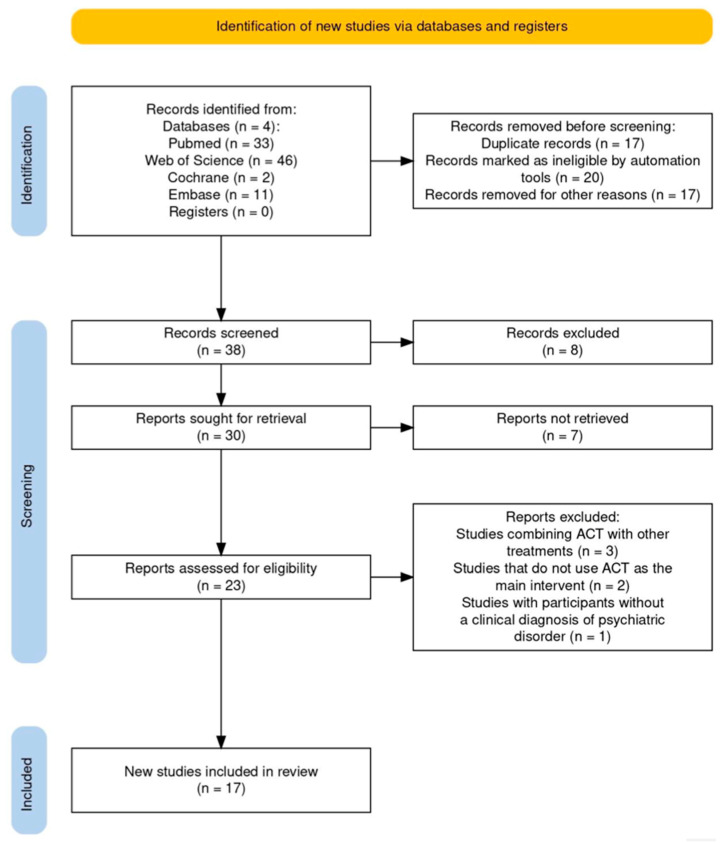
PRISMA 2020 flow diagram of evaluated studies.

Validated search strategies were independently developed and internally peer-reviewed by 2 reviewers (DL, GM) to warrant methodological rigor and transparency in the analysis of the efficacy of ACT on social functioning in adult psychiatric participants. The latter underwent iterative refinement, which included the addition of Boolean operators (AND, OR), keyword synonyms, and controlled vocabulary (e.g., MeSH) fields, with the goal of achieving balance between sensitivity and specificity, given the target of our review towards ACT-specific mechanisms. The selection procedure followed the PRISMA approach (identification, screening, eligibility, and composition) and is presented in Figure 1. Title, abstract, and full-text screening against eligibility criteria for studies related to ACT’s effect on social functioning was completed by both reviewers. Furthermore, independent data extraction and cross-validation were performed to minimize bias (publication, language, and time-lag bias). Data extracted were study design, number of participants, demographic characteristics of the participants, psychiatric disorders, intervention description, and outcomes specifically related to social functioning, such as psychological flexibility, acceptance, defusion, mindfulness, and value-based behavior change. To guarantee that applicable, extractable, and analyzable data could be found during the screening and data extraction process, a multi-step inter-rater reliability protocol was applied. All titles, abstracts, and full texts were reviewed independently by both reviewers. In addition to the Kappa statistic, with >0.61 being considered substantial agreement, raw percentage agreement was also determined to yield a broader perspective of agreement. For each stage, a calibration exercise was performed on a sample of the articles before the screening itself started to align interpretations of the eligibility criteria. We conducted regular consensus meetings during both attenuation and extraction to reconcile any differences in study inclusion or data extraction. In case of disagreement, a third reviewer (RSC) served as an adjudicator to reach a final decision. This procedure guarantees that all propriety decisions were transparent, clearly documented, and methodologically appropriate. Data extraction was also conducted in duplicate and cross-validated to minimize bias (e.g., publication, language, and time-lag bias). Data were systematically recorded using a custom-designed Excel template to ensure consistency with inclusion/exclusion criteria and enable tagging, sorting, and resolution of discrepancies. A thematic synthesis approach was employed to structure the final dataset, with an emphasis on therapeutic components relevant to ACT’s impact on social connectedness and recovery.

Extracted data included study design and methodology; sample size and demographic characteristics; psychiatric diagnosis; detailed ACT intervention characteristics; outcomes related to social functioning, including psychological flexibility, acceptance, cognitive defusion, mindfulness, and value-based behavioral change. This review has been registered on Open OSF (n) 2ZAGTand adheres to PRISMA-S 2021 reporting guidelines. A completed PRISMA-S checklist and the full search strategies for all databases are available in the Supplementary Material.

#### 2.4.2. Data Synthesis

Themes were inductively generated using a thematic synthesis approach, identifying recurring patterns across psychiatric diagnoses, ACT intervention characteristics, and targeted psychological mechanisms. This method enabled the extraction of key therapeutic domains, including acceptance, cognitive defusion, mindfulness, and values-based action. Considering the heterogeneity in study designs, patient populations, and outcome measures related to social functioning in psychiatric disorders, a narrative synthesis was employed to integrate findings across diverse methodologies. This combined magnification model allowed for the evaluation of ACT’s overall effectiveness by aggregating data from varied research designs and diagnostic groups. The narrative synthesis facilitated the identification of core psychological processes underpinning ACT-related change, specifically acceptance, defusion, mindfulness, and value-driven action. Studies were systematically coded by psychiatric diagnosis, intervention features, and measured outcomes related to both social and psychological domains. This framework enabled a nuanced examination of how different ACT components influence interpersonal functioning. Themes were developed by mapping recurrent ACT mechanisms (e.g., acceptance, values) to social functioning outcomes across diagnostic categories. The quality of evidence was assessed using predefined criteria, with studies categorized by methodological rigor, sample characteristics, and the degree to which outcome measures aligned with ACT’s theoretical framework. A multidisciplinary team of reviewers independently analyzed and interpreted the data, engaging in regular consensus meetings to minimize interpretative bias. The trustworthiness of thematic interpretations was enhanced through this collaborative and iterative process. Overall, the synthesized findings offer balanced and multidimensional insights into the potential of ACT as a recovery-oriented intervention to improve social functioning in psychiatric populations, while also outlining key directions for future research and clinical application.

### 2.5. Subgroup and Sensitivity Considerations

Due to the heterogeneity of study populations, intervention formats, and outcome measures, a formal subgroup or sensitivity analysis was not statistically feasible. However, we performed a structured narrative comparison across diagnostic groups (e.g., psychosis, PTSD, GAD), ACT delivery formats (individual, group, digital), and levels of methodological quality (low vs. moderate vs. high risk of bias). The synthesis highlights how ACT’s effects on social functioning may vary depending on psychiatric diagnosis and intervention characteristics. Additionally, results from high-risk studies were interpreted cautiously and did not substantially alter the overall thematic conclusions. This narrative triangulation approach served as a form of implicit sensitivity analysis to enhance the robustness of our findings.

## 3. Results

A systematic literature search in four electronic databases, PubMed (*n* = 33), Web of Science (*n* = 46), Cochrane (*n* = 2), and Embase (*n* = 11), resulted in a total of 92 records. At prescreening, 54 records were excluded (17 as duplicates, 20 through automated tools, and 17 for other reasons). This resulted in 38 papers being selected for review according to titles and abstracts. Eight of these were rejected because they were not relevant or the methodology was not appropriate. Thirty reports were identified and retrieved, seven of which could not be found with exhaustive searches (e.g., emailing corresponding authors, consulting libraries, exploring open-access sources, checking institutional resources, and using research networks). Based on the screening phase (23 articles), 6 studies were excluded (3 combined ACT with other therapies, 2 did not report ACT as the primary intervention, and 1 enrolled subjects without a clinical diagnosis of mental disorders). A total of 17 studies satisfied the predefined inclusion criteria and were incorporated in the final synthesis of this systematic review and are summarized in Table 3.

### 3.1. Assess Quality of Included Studies—Risk of Bias

**Figure 2:**
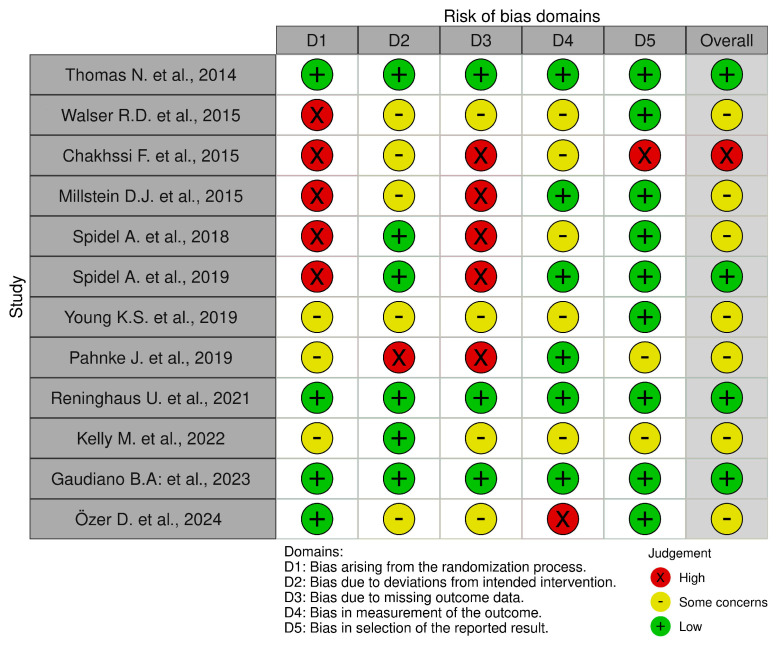
Risk of bias (RoB) in included RCT studies [43,44,45,47,49,50,51,53,54,55,56,57].

The risk of bias in controlled studies was assessed using a revised Cochrane risk of bias (RoB 2) tool [60], which comprises the following five domains: (i) bias arising from the randomization process; (ii) bias due to deviations from the intended intervention; (iii) bias due to missing outcome data; (iv) bias in the measurement of the outcome; and (v) bias in the selection of the reported result (Figure 2).

**Figure 3:**
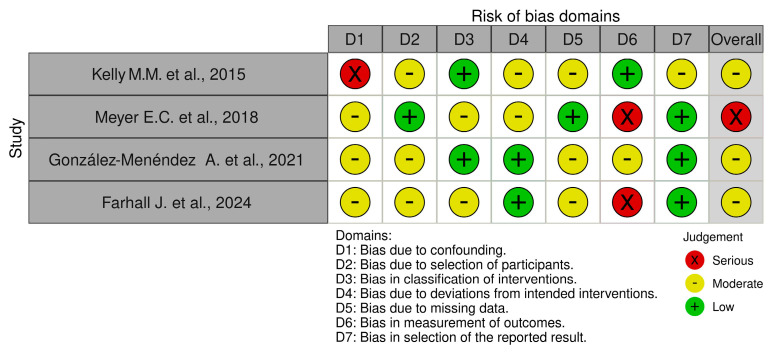
Cochrane risk of bias in non-randomized studies of interventions (ROBINS-I) [46,48,52,59].

The risk of bias in non-randomized studies of interventions (ROBINS-I) tool [61] comprises the following seven domains: (i) bias due to confounding; (ii) bias in selection of participants into the study; (iii) bias in classification of interventions; (iv) bias due to deviations from intended interventions; (v) bias due to missing outcome data; (vi) bias in measurement of the outcome; and (vii) bias in the selection of the reported results (Figure 3).

**Figure 4:**
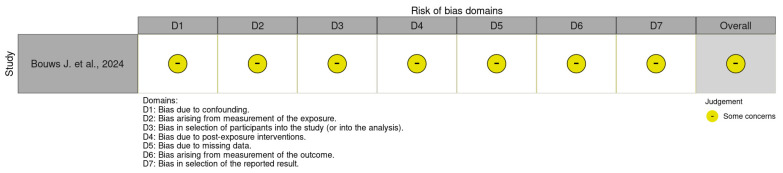
Cochrane risk of bias in non-randomized studies of exposures (ROBINS-E) [58].

Further, the risk of bias in non-randomized studies of exposures (ROBINS-E) tool [62] comprises the following seven domains: (i) bias due to confounding; (ii) bias arising from measurement of the exposure; (iii) bias in selection of participants into the study (or into the analysis); (iv) bias due to post- exposure interventions; (v) bias due to missing data; (vi) bias arising from measurement of the outcome; and (vii) bias in the selection of the reported result (Figure 4).

We identified only 4 studies with a low risk of bias, 11 with a moderate risk of bias, and 2 with a critical/high risk of bias and weak methodologies. Studies at low risk of bias were randomized control trials [43,50,53,56] that resulted in robust results due to the clear randomization process. The control of deviations from the intended intervention had an impact on studies analyzed due to the effectiveness and safety of the planned intervention and ensuring the internal validity of the studies. Strict adherence to the protocol enhanced motivation, understanding, and access to intervention to ensure the compliance of participants. Finally, analyses included all participants in the study, as originally assigned. Moderate risk of bias studies [44,46,47,49,51,52,54,55,57,58,59] were randomized control trials and non-randomized control trial studies. The common sources of bias identified among the 11 studies with a moderate risk were confounding factors. Most studies had limits due to insufficient strategies for controlling confounding factors, which could have influenced the outcomes. Some studies [44,49,51,54] presented their data in different ways, which raised concerns about transparency or completeness, and others used analytical methods that might introduce bias. Variability in data collection methods, such as inconsistent measurement tools, introduced bias. Even if these studies provide useful insights, their findings should be interpreted with caution, particularly where limitations about bias might influence the results, e.g., two critical/high-risk bias studies. The first was a non-randomized study of interventions [48] and the other was a randomized control study [45]. In Chakhssi’s study [45], the lack of random allocation of participants to groups provided a significant risk of bias. Without randomization, the groups being compared were not equivalent. This imbalance could influence the outcomes and lead to confounding; the effects observed may be due to casual differences rather than the intervention or exposure of interest. Moreover, missing outcome data can lead to biased estimates of the effect. In non-randomized studies, missing data could arise from loss to follow-up, non-response, or incomplete records. The extent and reasons for missing data should be carefully assessed, and appropriate methods such as multiple imputation or sensitivity analyses should be employed to address this bias. The study should also report the proportion of missing data and consider how its absence may impact the study’s conclusions [45]. Finally, Meyer’s study [48] presents a risk of bias in the selection of reported outcomes based on their statistical significance or the direction of results rather than a pre-specified analysis plan. This could be particularly problematic in non-randomized studies if the analysis plan is not registered or if selective reporting occurs, where only favorable results are reported. To minimize this risk, it is critical to predefine the outcomes and analysis methods in a protocol and to transparently report all results, including those that are non-significant or adverse. The consistency between the planned and reported outcomes should be documented [48]. These results are highly susceptible to bias, which significantly limits their credibility and the extent to which their findings can inform conclusions. While in Meyer’s study [48], there was no clear randomization process, the randomization process minimized selection bias. Even if this process is performed correctly, the risk of bias can still arise if the process is not adequately blinded or if the allocation sequence is not random. In the study, outcome data was missing, and the data analysis was not complete. This led to biased estimates of the treatment effect. It is important to assess the extent of missing data, the reasons for it, and the methods used to handle it in the analysis to determine the potential impact on the study’s findings.

### 3.2. Efficacy of ACT in Improving Social Support

Low social support is a critical determinant of both physical and mental health outcomes. Research consistently shows that individuals with low social support face higher risks of mortality, depression, and suicidal ideation, as well as poorer physical health due to the neglect of health-related behaviors like diet, exercise, and adherence to medical advice. By enhancing social connections, ACT demonstrates significant potential in addressing these challenges and improving overall well-being. Studies have shown that ACT interventions are effective in increasing social support across various populations. For example, in veterans with PTSD, ACT improved feelings of connection and reduced depression and suicidal ideation, helping individuals reconnect with strained social networks [47,54]. Similarly, ACT reduced self-stigma and increased psychological flexibility in individuals with chronic psychosis, leading to better social functioning and stronger support systems [58]. These findings highlight how ACT addresses psychological barriers to social engagement, allowing individuals to rebuild meaningful relationships. In patients with generalized anxiety disorder (GAD), ACT has been shown to improve interpersonal relationships through enhanced mindfulness and reduced interpersonal difficulties. Participants in an ACT-based therapy reported improved social ties, stemming from their increased ability to manage anxiety and engage more actively in social relationships [44]. Similarly, ACT-based interventions for psychosis and trauma demonstrated improved emotion regulation, enabling participants to sustain and enhance their social connections. This suggests that ACT helps individuals better manage emotional challenges, thereby fostering more robust social support systems [49]. The neural mechanisms underlying ACT’s impact on social functioning have also been explored. A study on individuals with social anxiety disorder (SAD) found that changes in brain connectivity, particularly in response to affect labeling, correlated with improved social support outcomes. Participants who developed better emotional regulation skills were able to form more meaningful and supportive relationships, suggesting that ACT promotes social functioning by enhancing both emotional and neural flexibility [51]. 20 By reducing avoidance, fostering acceptance, and promoting mindfulness, ACT equips individuals to navigate their social worlds with greater empathy and attentiveness. This leads to stronger social bonds, improved emotional resilience, and better psychological well-being, making ACT a valuable intervention for enhancing social support across diverse mental health populations.

### 3.3. The Role of ACT in Improving Psychiatric Symptoms

ACT has shown significant potential in reducing psychiatric symptoms across a wide range of mental health disorders. This therapeutic approach builds psychological flexibility, allowing individuals to accept their thoughts and emotions without judgment and engage in value-driven actions. Studies demonstrate that ACT not only reduces the severity of symptoms such as anxiety, depression, and psychosis but also enhances patients’ ability to manage their mental health effectively, promoting better overall well-being [43,44,45,46,47,48,50,51,52,53,54,56,57,58,59,63].

### 3.4. Anxiety, Depression, and PTSD

ACT is recognized for its efficacy in treating anxiety and depressive disorders by addressing maladaptive patterns such as avoidance and rumination. For depression, ACT fosters acceptance and values-based engagement, helping individuals regain motivation and purpose. Similarly, for anxiety disorders and PTSD, ACT reduces distress by promoting psychological flexibility, allowing individuals to confront trauma related thoughts and emotions without avoidance. For example, a randomized study on veterans with PTSD and alcohol use disorder demonstrated that ACT significantly reduced symptoms in both areas, promoting healthier coping strategies [48]. Additionally, ACT’s utility in veterans with PTSD extends to smoking cessation, with participants reporting reduced PTSD-related symptoms and improved control over intrusive thoughts [46].

### 3.5. Psychotic Symptoms

ACT has been extensively studied in the context of psychosis. In inpatient settings, it has been shown to reduce the distress associated with psychotic symptoms such as delusions and hallucinations. A randomized trial found that ACT enabled participants to develop a new relationship with their symptoms, reducing interference with daily life and promoting psychological flexibility [56]. Similarly, Thomas et al. [43] demonstrated that ACT improved general functioning and reduced distress from psychotic symptoms. These findings suggest that ACT is an effective intervention for managing severe psychiatric symptoms, particularly when integrated into recovery-oriented group interventions [59].

#### 3.5.1. Complex and Early Psychosis

ACT has also shown promise in addressing early psychosis and complex cases. A qualitative study of individuals at risk of developing psychosis highlighted significant improvements in managing distressing thoughts and hallucinations, with ACT fostering acceptance and reducing symptom severity [58]. Additionally, a randomized trial investigating ACT for early psychosis in daily life found that it effectively reduced symptom intensity and frequency, improving quality of life [53]. The potential of ACT in early intervention is further supported by its flexibility in remote formats, as demonstrated by an online ACT program that reduced hallucinations and delusions while enhancing daily functioning [57].

#### 3.5.2. Resistant and Comorbid Conditions

In cases of treatment-resistant conditions, such as personality disorders or co-occurring mental health challenges, ACT has proven efficacious. A study on patients with personality disorders reported significant reductions in symptoms of emotional instability, anxiety, and depression, with ACT enabling better acceptance of negative emotions and improved overall functioning [45]. Similarly, ACT has been shown to effectively address symptoms in individuals with co-occurring PTSD and trauma, improving emotional regulation and reducing symptom severity, even in those with severe trauma histories [50]. In summary, ACT’s focus on psychological flexibility and acceptance proves highly effective in addressing diverse psychiatric symptoms. By equipping individuals with tools to manage distress and engage in meaningful actions, ACT facilitates symptom reduction and fosters long-term resilience, even in complex or treatment-resistant cases.

## 4. Discussion

Some studies showed that ACT was used for reducing some psychiatric symptoms as well as delusions, hallucinations, suicidal ideation, and psychological inflexibility, while simultaneously enhancing psychological flexibility, emotional regulation, and mindfulness [54,64,65]. However, these findings are not actually consistent to define the treatment effective due to limitations of diagnosis heterogeneity, small sample sizes, high risk of bias, and absence of control group treated by another therapy in comparison to define a greater efficacy. These findings have important clinical implications. Improvements in social functioning are not merely ancillary benefits but core outcomes that can significantly enhance the prognosis of psychiatric conditions. Enhanced social connectedness has been linked to reduced symptom severity, increased treatment adherence, and improved overall quality of life. By fostering psychological flexibility and reconnecting individuals with their social environments, ACT contributes to a more comprehensive recovery model that prioritizes not only symptom relief but also social reintegration and functional autonomy. This underscores the value of integrating ACT into routine clinical practice for individuals facing social withdrawal or interpersonal dysfunction across diagnostic categories. ACT’s role in enhancing social support is particularly noteworthy. Notably, enhanced social functioning is associated with a wide range of clinical benefits, including reduced symptom burden, improved quality of life, lower relapse rates, and stronger therapeutic engagement. It reconnects individuals with their social networks and strengthens their capacity for meaningful relationships by fostering mindfulness and emotional regulation. For instance, Xu et al. [64] demonstrated that mindfulness, a core component of ACT, enhanced social functioning and emotional resilience in adolescents with depression and trauma, while Wharton et al. [66] observed improvements in psychological functioning and emotional regulation following ACT interventions. Compared with traditional cognitive behavioral therapy, ACT exhibits unique advantages. While a meta-analysis found no significant differences in individual treatment outcomes, group-based ACT demonstrated superior success rates and lower dropout rates, particularly in the treatment of somatic and psychic conditions [67]. However, ACT’s mindfulness-based strategies may present challenges for individuals who struggle with abstract concepts or find difficulty engaging in metaphors, a limitation that CBT may address more effectively in certain cases [68,69]. Despite the promising findings, several gaps in the literature merit discussion. Many studies rely on small sample sizes or single-site interventions, which limit the generalizability of their findings. For example, while Kelly et al. [54] and Khoramnia et al. [65] demonstrate ACT’s efficacy in enhancing social functioning and emotional regulation, these studies focus on specific populations, such as veterans or individuals with social anxiety, and may not fully capture the therapy’s broader applicability across diverse cultural and socioeconomic contexts. Furthermore, reliance on self-reported outcomes in many studies introduces potential bias, underscoring the need for objective measures and multi-method approaches in future research [66]. The long-term durability of ACT’s benefits also requires further exploration. While Xu et al. [64] and Ferreira et al. [67] report sustained improvements in social functioning and symptom reduction, longitudinal studies with extended follow-up periods are necessary to confirm these outcomes and identify factors that influence treatment retention and effectiveness over time. Additionally, while ACT has been shown to be effective for a range of conditions (including psychosis, autism, and substance use disorders) it is unclear whether its efficacy varies significantly across different psychiatric profiles or in combination with other therapeutic modalities, such as trauma-focused CBT or medication [70,71]. Expanding studies to include diverse cultural and socioeconomic populations would improve the generalizability of findings and identify any cultural adaptations needed for ACT’s efficacy. Additional research into the neural and cognitive mechanisms underlying ACT’s effects would provide a deeper understanding of how it promotes psychological flexibility and reduces psychiatric symptoms [64,69]. Beyond the clinical insights, it is essential to reflect on the methodological rigor and limitations of the current evidence base, which may influence the interpretation and generalizability of our findings. The overall quality of the included studies varied, with only a limited number rated as low risk of bias. These higher quality studies contributed more robust evidence, whereas those with moderate or high risk presented limitations such as inadequate randomization, confounding, and missing outcome data. Although studies were coded by methodological quality, no formal subgroup or sensitivity analyses were conducted to test whether study quality affected outcome patterns—this represents a key area for future reviews. Moreover, the review was limited to English-language peer-reviewed articles, potentially introducing language and publication bias. The lack of formal PRESS peer review of the search strategy also represents a methodological constraint. While internal peer checking was conducted, future systematic reviews would benefit from externally validated search protocols. Lastly, although the synthesis was guided by the thematic clustering of ACT mechanisms across studies, further work is needed to expand the granularity of synthesis and strengthen causal inferences. An important consideration when interpreting the results is the variability across study populations and intervention characteristics. Included studies differed in diagnostic categories, age groups, clinical settings (e.g., inpatient, outpatient, community-based), and cultural backgrounds. Furthermore, the implementation of ACT varied in terms of delivery format (group vs. individual), session duration and frequency, and therapist training or fidelity monitoring. These differences may have contributed to heterogeneity in treatment outcomes and limit the extent to which findings can be generalized across contexts. Future research should aim to systematically compare these variations and explore their moderating effects to identify for whom and under what conditions ACT is most effective. Finally, it is important to note how study quality may have influenced the observed findings. In our synthesis, studies rated as low risk of bias more consistently reported improvements in both social functioning and psychological outcomes, while studies with higher risk presented more variable or limited effects. Although we did not conduct a formal stratified analysis, this pattern suggests that methodological rigor plays a key role in determining the strength of reported ACT effects.

### 4.1. Limitations

Moreover, several limitations may have affected the robustness of this review. First, by including only English-language peer-reviewed publications, there is a potential for both language and publication bias, as studies with non-significant or null results are less likely to be published or indexed. Second, although internal quality control of the search strategy was performed, the lack of external validation (e.g., PRESS review) may have introduced search bias or missed relevant records. Third, the use of narrative synthesis, while appropriate given the heterogeneity of included studies, inherently limits the ability to draw causal inferences or conduct statistical comparisons. Future reviews might benefit from integrating meta-analytic components where feasible and adopting formal frameworks (e.g., GRADE) to assess confidence in evidence across themes.

Also severe methodological and conceptual limitations were found. One of the primary limitations pertains to the highly heterogeneous nature of the studies, including diagnosis, impact, and outcome. As the present review only contains one or two studies for each diagnostic category, it does not function as the synthesis of a broad body of the literature on one disorder or treatment; rather, it represents the collection of a relatively small number of isolated studies on various psychiatric illnesses. Therefore, such wide variability of conditions limits the credibility of the cross-study comparisons and significantly undermines the capacity for generalized conclusions about the efficacy of ACT for a particular psychiatric population. In addition, most of the included studies are underpowered due to the small sample size and lack of randomization and have a high or moderate risk of bias when assessed through established tools. More specifically, common methodological pitfalls of the identified studies relate to the non-randomized and biased conditions, confounding, lack of blinding, and the use of subjective or self-reported outcomes. The critical aspect of these limitations is the absence or inadequate control group in most of the studies included. It means that any level of inference about the causality of the observed association is precluded upon these studies, as the real efficacy of the ACT cannot be adequately assessed. Therefore, the high risk of observation bias does not discredit the reliability of the results but instead emphasizes the inability to interpret them as causal evidence. For most studies, there is an inadequate number of blinding approaches and no reporting of objective outcomes, or the outcomes are measured with established scales or tests, and as such, their findings on social and psychiatric impacts lose credibility. Moreover, such limitations as high attrition rates in several primary studies undermine the robustness of the legitimacy of such claims. These limitations, in combination with the lack of longitudinal data and limited representation of diverse or underrepresented populations, significantly weaken the capacity of this systematic review to provide strong actionable insights into the efficacy of ACT for improving social functioning in adults with psychiatric disorders. Moreover, substantial variability in the tools used to assess social functioning across studies may have affected the comparability and robustness of findings.

Therefore, this review should not be approached as the statement of the definitive effect of ACT but rather as the broad and comprehensive mapping of the highly diverse inconsistent literature. For the field of future research, the identified limitations strongly suggest the need for larger control-group-based studies that will encompass homogeneous sets of diagnoses and measure outcomes objectively, using established scales. Acknowledging these limitations is crucial for interpreting the present findings and for guiding future research efforts toward more methodologically robust and inclusive investigations.

### 4.2. Strengths

Despite these limitations, the review offers valuable contributions to the literature. A key strength lies in its specific emphasis on social functioning outcomes, an area often overlooked in psychiatric research. By integrating both symptom reduction and functional improvement, this review highlights the clinical relevance of ACT’s dual benefits, supporting a more holistic model of recovery. Furthermore, the systematic approach adopted—including a comprehensive search strategy and the application of validated risk of bias assessment tools—enhances the methodological transparency and reliability of the synthesis. While the findings must be interpreted with caution, they offer a critical mapping of the current evidence base and outline key priorities for future more methodologically rigorous investigations.

## 5. Conclusions and Future Directions

To conclude, this systematic review has documented and quantified considerable limitations across the existing body of evidence. No firm conclusion can be drawn regarding the efficacy of ACT on social functioning in psychiatric patients because of diagnosis heterogeneity, small sample sizes, and a high risk of bias. The poor operationalization lacking robust control groups, the primary focus on self-reported outcomes, and the generally poor methodological quality introduce an excessive risk of bias. In other words, our findings are preliminary and descriptive, and their purpose is to illustrate the current state of evidence, which is fragmented and marked with low quality, rather than provide evidence for or against the use of ACT in this context. Larger, more representative, randomized, and blinded trials that focus on more homogeneous diagnostic groups with objective outcomes are needed. Without more robust research characterized by higher methodological rigor and more homogenous study samples, the true efficacy and specific clinical utility of ACT in the psychiatric patient population will remain unknown.

## Figures and Tables

**Table 1:** PICO framework and study inclusion criteria.

PICO Element	Criteria/Description
Population	Adults (≥18 years) with psychiatric diagnoses (e.g., anxiety, depression, PTSD, psychosis, ASD, insomnia)
Intervention	Acceptance and commitment therapy (ACT), delivered individually, in group, or digitally
Comparison	CBT, psychoeducation, communication training, treatment-as-usual, or no treatment
Outcomes	Improvements in psychiatric symptoms, social functioning, psychological flexibility, QoL
Inclusion	Peer-reviewed studies (RCTs or controlled trials), published 2014–2024, English language
Exclusion	Studies without ACT-based intervention, non-peer-reviewed sources, pediatric populations
Data extracted	Study design, sample size, diagnosis, intervention type, outcome measures, main results

This table summarizes the core components of the PICO framework (population, intervention, comparison, outcome) used to structure the search strategy and define eligibility criteria. It outlines the target population, the type of interventions included, comparator conditions, and relevant outcomes. The inclusion criteria were designed to ensure methodological transparency and support the clinical relevance of the findings.

**Table 2:** Core search terms and Boolean strategy across databases.

PICO Element	Keywords/Terms Used
Population	“psychiatric disorder”, “mental health”, “psychosis”, “depression”, “anxiety”, “PTSD”, “insomnia”, “autism spectrum disorder”
Intervention	“Acceptance and Commitment Therapy”, “ACT therapy”, “third wave therapy”
Comparison	“cognitive behavioral therapy”, “CBT”, “psychoeducation”, “treatment as usual”, “control group”, “communication training”
Outcomes	“social functioning”, “psychological flexibility”, “interpersonal relationships”, “quality of life”, “emotional regulation”, “psychiatric symptoms”

The above keywords were adapted into database-specific syntax (e.g., Boolean operators, truncation, field tags) for each platform. The full PubMed strategy is included in this manuscript; other search strings are available upon request.

**Table 3:** Summary of studies included in the research.

Author/Location/Country	Study Design	Population/Diagnosis	Sample Size/Participants’ Characteristics	Outcome Measures	Intervention	Major Findings	Effect Size and Certainty of Evidence
Thomas N et al., 2014. [43] Location: Metropolitan Melbourne, Victoria. Country: Australia.	Randomized Control Trial	Patients with drug-resistant chronic psychotic symptoms	Sample size: 53 Age: 18–65 years Sex: not specified	PANSS; PSYRATS; PDI; SFS; SDS; AAQ; RSQ; WAIS-III; RBANS; CSQ8	ACT Protocol	Findings suggest that ACT is an effective treatment for reducing distress and disability associated with psychotic symptoms.	Effect size: The sample size calculation is based on detecting an effect size of d = 0.55 or greater for between-group differences in overall mental state, with 80% power and α = 0.05. Previous trials reported effect sizes around d = 0.60. Certainty of evidence: moderate.
Millstein D.J. et al., 2015 [44] Location: Boston, Massachusetts. Country: United States	Randomized Control Trial	Patients with GAD	Sample size: 81 Age: mean age of participants was 32.92 years Sex: overall, 65.4% were female (*n* = 53) and 34.6% male (*n* = 28)	ADIS-IV, IIP-SC, FFMQ	Acceptance Based Behavior Therapy (experimental)/Applied Relaxation (control)	Mindfulness in GAD may help ameliorate interpersonal difficulties.	Effect size: Both ABBT and AR led to large reductions in interpersonal problems (pre-post effect sizes: ABBT d = 0.98, AR d = 0.84). There were no significant differences between treatment groups in primary or secondary outcomes (e.g., pre-treatment GAD severity F(1,79) = 0.37, *p* = 0.55; pre-treatment interpersonal problems F(1,77) = 0.24, *p* = 0.62)). Certainty of evidence: moderate.
Chakhssi F. et al., 2015 [45] Location: two specialized day hospital sites in different towns (Apeldoorn and Eindhoven). Country: The Netherlands.	Randomized Control Trial	Patients with personality disorders	Sample size: 81 Age: mean age 32.98 years Sex: ACT group: 85.0% female (*n* = 51), 15.0% male (*n* = 9); CBT-TAU group: 76.2% female (*n* = 16), 23.8% male (*n* = 5).	AAQ-II; WHOQOL	ACT Protocol	ACT is an effective treatment for individuals with personality pathology.	Effect size: Within-group effect sizes for primary and secondary outcomes were small to moderate (ACT d = 0.31, CBT-TAU d = 0.06). A significantly higher proportion of ACT participants showed reliable improvement on the primary outcome compared with CBT-TAU (χ^2^ = 4.800, *p* = 0.029). Certainty of evidence: low.
Kelly M. et al., 2015 [46] Location: Edith Nourse Rogers Memorial Veterans Hospital, Bedford, Massachusetts. Country: United States	Uncontrolled Pilot Study	Veterans with PTSD	Sample size: 19 Age: mean age was 56.0 years Sex: all participants were male (100%).	PTSD Symptoms; Treatment Adherence; Quit Attempts; Readiness to Change; Treatment Acceptability	ACT Protocol	ACT appears to be a promising smoking cessation treatment for veterans with PTSD.	Effect size: Significant reductions in number of cigarettes smoked per day were observed, F(3, 54) = 20.12, *p* < 0.001, with a 62% reduction at end of treatment and 43% at three-month follow-up. PTSD symptoms also significantly decreased over time, F(3, 54) = 10.43, *p* < 0.001, remaining reduced at one- and three-month follow-up (*p* = 0.001). Smoking urges related to both pleasure and negative effect significantly decreased from baseline to post-treatment and follow-up (all *p* < 0.05). Certainty of evidence: low.
Walser R.D. et al., 2015 [47] Location: Veterans Affairs Palo Alto Health Care System Country: United States	Randomized Control Trial	Veterans with depression and suicidal ideation	Sample size: 981 Age: mean age: 50.5 years Sex: 76% male (*n* = 741), 22.6% female (*n* = 222), 1.8% not reported	BDI-II; SUICIDAL IDEATION; FMMQ; AAQ-II	ACT for Depression Protocol	Depression severity and odds of suicidal ideation decrease during treatment.	Effect size: Significant reductions in depression severity were observed (mean BDI-II decreased from 30.8 to 20.0), with a time effect of b = 10.52, *p* < 0.001. Patients with suicidal ideation at baseline had a greater reduction in depression severity after adjusting for acceptance and mindfulness (interaction effect b = 2.81, *p* = 0.001). Certainty of evidence: moderate.
Meyer E.C. et al., 2018 [48] Location: Country: Stati Uniti, USA	Uncontrolled Pilot Study	Veterans with PTSD and alcohol use disorders	Sample size: 29 veterans (67% of 43 enrolled participants) Age: not reported Sex: not specified	CAPS-5; PCL-5; SCID-5; AUDIT; WHODAS 2.0; WHOQOL-BREF; PHQ9; AAQ-II; BEAQ; DAST	ACT for PTSD-AUD	ACT for PTSD–AUD is feasible and promising for recovery.	Effect size: PTSD clinician-rated symptoms: *d* = 0.79; PTSD self-report symptoms: *d* = 0.96 post-treatment, *d* = 0.88 at follow-up; alcohol outcomes: mean *d* = 0.91 (range: 0.65–1.30); quality of life: *d* = 0.55–0.56; functional disability: *d* = 0.35 (post), *d* = 0.52 (follow-up); depressive symptoms: *d* = 0.50 (post), *d* = 0.44 (follow-up). Certainty of evidence: low.
Spidel A. et al., 2018 [49] Location: University of Montreal and UBC, group intervention Country: Canada	Randomized Control Trial	Patients with psychosis and trauma	Sample size: 50 (randomized), intervention group size not explicitly reported in abstract Age: not reported Sex: not specified	CERQ, BPRS-E, TSC-40, GAD-7, Service Engagement Scale, Feedback interview	ACT (experimental)/Treatment as Usual	ACT is a promising treatment for patients with psychosis and a history of trauma.	Effect size: not reported. Certainty of evidence: moderate.
Spidel A. et al., 2019 [50] Location: University of Montreal and UBC; group-delivered ACT Country: Canada	Randomized Control Trial	Patients with psychosis and childhood trauma	Sample size: 50 (randomized total sample; group sizes not specified in abstract) Age: not reported Sex: not specified	Not Specified	ACT (experimental)/Treatment as Usual	ACT training is promising regardless of trauma severity.	Effect size: not reported. Certainty of evidence: moderate.
Young KS, et al., 2019 [51] Location: University of California, Los Angeles Country: USA	Randomized Control Trial	Patients with SAD	Sample size: 50 (RCT), exact group allocations not specified in abstract. Age: not reported Sex: not specified	ADIS-IV; CSR; LSAS-SR; SIAS	ACT/CBT	Both therapies significantly reduced symptoms.	Effect size: not reported. Certainty of evidence: moderate.
González-Menéndez A. et al., 2021 [52] Location: Asturias, public mental health outpatient service. Country: Spain	Longitudinal Study	People with chronic psychosis	Sample size: 103 adult outpatients with chronic psychosis Age: M = 49.68 years (SD = 12.28) Sex: 60 males/43 females	ISMI; AAQ-II; SCS	ACT	ACT may attenuate self-stigma and improve social functioning.	Effect size: From PROCESS mediation analysis (bootstrap): PI → psychosis severity: B = 0.37, *p* = 0.0001; PI → self-stigma: B = 0.66, *p* < 0.00001; self-stigma → social functioning: B = –0.24, *p* = 0.039; social functioning → psychosis severity: B = –0.28, *p* = 0.007; PI → social functioning: B = –0.31, *p* = 0.009; indirect effect (PI → stigma + social functioning → severity): B = 0.279, BootSE = 0.103; 95% CI [0.089, 0.492]. Certainty of evidence: moderate.
Reininghaus U et al., 2021 [53] Location: Country: The Netherlands and Belgium	Multicenter Randomized Controlled Trial	Subjects with UHR and FEP	Sample size: 148 participants (71 in ACT-DL + TAU; 77 in TAU group) Age: 15–65 years Sex: not reported	CAARMS, GAF, SOFAS, SFS, ESM, BPRS, BNSS, PANSS	ACT-DL + TAU/TAU	ACT-DL is effective and supports mHealth in mental health.	Effect size: not available, this is a study protocol; no statistical results are reported. Certainty of evidence: very low.
Kelly M. et al., 2022 [54] Location: VA Bedford Healthcare System & University of Massachusetts Chan Medical School Country: USA	Randomized Control Trial	U.S. veterans with PTSD	Sample size: 40 (21 ACT-SS; 19 PCT) Age: not reported Sex: not specified	SCID-5; SAS-SR; MOS-SS; QLES-Q-SF; PCL-5; MAAS; AAQ-II; VLQ; CSQ-8; WAI-S	ACT-SS/Person-Centered Therapy	ACT-SS is feasible and shows positive preliminary outcomes.	Effect size: not reported in abstract; however, significant improvements were noted in social relationship quality, social/leisure activity engagement, PTSD symptoms, mindfulness, valued living, and experiential avoidance from baseline to post-treatment and at 3-month follow-up in the ACT-SS group but not in PCT. Certainty of evidence: moderate.
Pahnke J. et al., 2019 [55] Location: Karolinska Institutet, Psychiatric Outpatient Clinic, Stockholm Country: Sweden	Randomized Control Trial	Adults with autism and Asperger’s syndrome	Sample size: 9 of 10 adults diagnosed with ASD (5 male, 5 female; age 25–65 years) Age: not specificated. age range provided (25–65 years) Sex: 5 males, 5 females; 9 completers	PSS-14, SWLS, QOLI, AAQ, CFQ, CBAS, KSQS, KSQ-A, SDS	NeuroACT/Ordinary Care	NeuroACT may be beneficial for adults with autism with low quality of life.	Effect size: not reported numerically; significant reductions and increases reported for several outcomes: PSS (stress): *p* = 0.023 (pre→post); SWLS (life satisfaction): *p* = 0.021 (pre→3-month follow-up); depression, social disability, cognitive fusion, psychological flexibility: *p* < 0.05. Certainty of evidence: low.
Gaudiano B.A. et al., 2023 [56] Location: Brown University, Butler Hospital, Providence VA Medical Center, SUNY Upstate, Michigan State University, Boston University Country: USA	Randomized Control Trial	Patients with schizophrenia	Sample size: 46 inpatients with schizophrenia-spectrum disorders (23 ACT-IN + TAU; 23 TAM + TAU) Age: not reported Sex: not specified	SCID-5; MMSE; CSQ-8	ACT-IN/TAM/TAU	ACT-IN showed greater satisfaction and reduced suffering.	Effect size: Not specified in the abstract; significant but similar improvements across conditions on symptom, functioning, and mindfulness outcomes. Only ACT-IN showed greater reduction in distress. Rehospitalization risk was lower in ACT-IN group (hazard ratio implied: TAM had 3.76× greater risk) Certainty of evidence: moderate.
Özer D et al., 2024 [57] Location: online group-based intervention) Country: Turkey	Randomized Control Trial	Patients with schizophrenia and other psychotic disorders	Sample size: 65 individuals with early psychosis (randomized to ACT-IN [*n* = 33] vs. TAU [*n* = 32]) Age: not reported Sex: not reported	CEQ, BPRS, CORE, WHODAS-II, QLS, AAQ-II, ACAMS-R, VQ, THI, PANSS, SFAS	Online Group ACT	Online ACT reduces psychotic symptoms and hospitalizations.	Effect size: not specified numerically; abstract reports: ACT significantly reduced psychotic symptoms score from 128 to 104 (Z = 5.01) vs. TAU from 130 to 117 (Z = 4.88) Certainty of evidence: moderate.
Bouws J. et al., 2024 [58] Location: Country: The Netherlands and Belgium	Qualitative Study	Individuals at risk for psychosis or first psychotic episode	Sample size: 19 Age: not reported Sex: not reported	Semi-structured interviews	ACT Protocol	ACT is acceptable and promising for early stages of psychosis.	Effect size: not applicable, no quantitative outcomes reported. Certainty of evidence: very low.
Farhall J. et al., 2024 [59] Location: La Trobe University and public mental health services Country: Australia (Victoria)	Uncontrolled Pilot Study	Adults diagnosed with psychotic disorders	Sample size: 80 adults with psychosis enrolled; 39 completers (participated in ≥3 sessions) Age: not reported Sex: not reported	Questionnaire about recovery, CORE-10, SMQ, CFQ, AAQ-II	ACT Intervention	Recovery ACT groups are feasible, acceptable, and safe in public services.	Effect size: paired t-tests show significant pre-post increases: personal recovery, well-being, mindfulness, committed action → small effect sizes; experiential avoidance → small effect size; committed action → medium effect size. Certainty of evidence: low.

Legend: Positive and Negative Syndrome Scale (PANSS), Psychotic Symptom Rating Scales (PSYRATS), Peters Delusions Inventory (PDI), Social Functioning Scale (SFS), Sheehan Disability Scale (SDS), Acceptance and Action Questionnaire (AAQ), Recovery Style Questionnaire (RSQ), Wechsler Adult Intelligence Scale III (WAIS-III), Repeatable Battery for the Assessment of Neuropsychological Status (RBANS), Client Satisfaction Questionnaire-8 (CSQ-8), acceptance and commitment therapy (ACT), generalized anxiety disorder (GAD), Anxiety Disorders Interview Schedule-IV (ADIS IV), Interpersonal Problems –Short Circumplex (IIP –SC), Five Facet Mindfulness Questionnaire (FFMQ), acceptance-based behavior therapy (ABBT), applied relaxation (AR), generalized anxiety disorder (GAD), treatment-as-usual based on cognitive behavioral therapy (CBT-TAU), Acceptance and Action Questionnaire (AAQ-II), World Health Organization Quality of Life—BREF (WHOQOL), post-traumatic stress disorder (PTSD), Beck Depression Inventory (BDI-II), Freiburg Mindfulness Meditation Questionnaire (FMMQ), Clinician-Administered PTSD Scale for DSM-5 (CAPS-5), Posttraumatic Stress Disorder Checklist 5 (PCL-5), Structured Clinical Interview for DSM-5 (SCID-5), Alcohol Use Disorders Identification Test (AUDIT), World Health Organization Disability Assessment Schedule (WHODAS), Patient Health Questionnaire-9 (PHQ-9), Brief Evaluation of Anxiety and Depression Questionnaire (BEAQ), Drug Abuse Screening Test (DAST), alcohol use disorder (AUD), Cognitive Emotion Regulation Questionnaire (CERQ), Brief Psychiatric Rating Scale—Expanded Version (BPRS-E), Trauma Symptom Checklist-40 (TSC-40), Anxiety Disorders Interview Schedule for DSM-IV (ADIS-IV), Clinical Severity Rating (CSR), Liebowitz Social Anxiety Scale –Self-Report Version (LSAS-SR), Social Interaction Anxiety Scale (SIAS), cognitive behavioral therapy (CBT), Internalized Stigma of Mental Illness (ISMI), Social Comparison Scale (SAS), Ultra-High-Risk (UHR), First Episode Psychosis (FEP), The Comprehensive Assessment of At-Risk Mental States (CAARMS), Global Assessment of Functioning (GAF), Social and Occupational Functioning Assessment Scale (SOFAS), experience sampling method (ESM), Brief Psychiatric Rating Scale (BPRS), Brief Negative Symptom Scale (BNSS), treatment as usual (TAU), Social Adjustment Scale-Self Report (SAS-SR), Medical Outcomes Study Sleep Scale (MOS-SS), Quality of Life Enjoyment and Satisfaction Questionnaire Short Form (QLES-Q-SF), Mindful Attention Awareness Scale (MAAS), Valued Living Questionnaire (VLQ), Working Alliance Inventory-Short Form (WAI-S), Perceived Stress Scale (PSS-14), Satisfaction With Life Scale (SWLS), Quality of Life Inventory (QOLI), Cognitive Failures Questionnaire (CFQ), Czech Brain Aging Study (CBAS), Knowledge and Skills Questionnaire—Short Form (KSQ-S), Mini-Mental State Exam (MMSE), Credibility and Expectancy Questionnaire (CEQ), Clinical Outcomes in Routine Evaluation (CORE), World Health Organization Disability Assessment Schedule-II (WHODAS-II), Quality of Life Scale (QLS), Action Questionnaire-II (AAQII), Acceptance and Commitment Therapy Adherence Measure Scale-Revised (ACAMS-R), Valuing Questionnaire (VQ), Treatment History Interview (THI), Acceptance and Commitment Therapy for Individuals with Psychosis (ACT-IN), treatment as usual model (TAM), Social Functioning Assessment Scale (SFAS), Clinical Outcomes in Routine Evaluation-10 (Core-10), Acceptance and Commitment Therapy for Individuals with Psychosis (ACT-IN), Beck Depression Inventory—Second Edition (BDI-II), Clinical Outcomes in Routine Evaluation—10 items (CORE-10), Client Satisfaction Questionnaire—8 items (CSQ8), Generalized Anxiety Disorder—7 item scale (GAD-7), Suicidal Ideation (IDEATION), Inventory of Interpersonal Problems—Short Circumplex (IIP-SC), Knowledge and Skills Questionnaire—Autism Version (KSQ-A), Knowledge and Skills Questionnaire—Short Form (KSQS), PTSD Checklist for DSM-5 (PCL-5), Patient Health Questionnaire—9 items (PHQ9), Perceived Stress Scale—14 items (PSS-14), Quality of Life Enjoyment and Satisfaction Questionnaire—Short Form (QLES-Q-SF), Social Motivation Questionnaire (SMQ), World Health Organization Quality of Life—Brief Version (WHOQOL-BREF).

## Data Availability

Not applicable.

## Supplementary Materials

The following supporting information can be downloaded at: https://www.mdpi.com/article/10.3390/healthcare13131587/s1. Reference [72] is cited in the supplementary materials.

## Abbreviations

The following abbreviations are used in this manuscript:

ACT	Acceptance and commitment therapy
CBT	Cognitive behavioral therapy
RCT	Randomized clinical trials
PTSD	Post-traumatic stress disorder
GAD	Generalized anxiety disorder
